# 06 Flat foot impairs physical activity in children with juvenile idiopathic arthritis

**DOI:** 10.1093/rheumatology/keac496.002

**Published:** 2022-10-07

**Authors:** Hanene Lassoued Ferjani, Yasmine Makhlouf, Lobna Ben Ammar, Wafa Triki, Dorra Ben Nessib, Kaouther Maatallah, Dhia Kaffel, Wafa Hamdi

**Affiliations:** Department of Rheumatology, Kassab Institute of Orthopedics, Tunis, Tunisia

## Abstract

**Background:**

Children with juvenile idiopathic arthritis (JIA) are less active compared with the general population due to pain and deformities, mainly of the lower limbs. Indeed, only 23% of children with JIA meet the public health recommendations of one h of moderate to vigorous physical activity daily [1]. In this context, foot involvement in JIA is a considerable limiting cause of physical activity.

**Objectives:**

Our study aimed to investigate the types of static foot disorders that impede physical activity in children with JIA.

**Methods:**

We conducted a cross-sectional study of patients with JIA according to the revised ILAR criteria. Socio-demographic and disease-related data were recorded. All patients underwent podoscope examination for varus or valgus deformity of the hindfoot and plantar footprint abnormality (flat foot or hollow foot). Patients with ankle or foot involvement due to congenital malformation or any other cause besides JIA were excluded. The impact of foot involvement on physical activity was assessed by the Oxford ankle foot questionnaire for children (OxAFQ-C). A higher score represents better functioning. We looked for the effect of foot abnormalities and their impact on physical activity.

**Results:**

A total of 23 patients were collected. The mean age was 12.7 ± 3 [6–18] years and the mean age at diagnosis was 9.3 ± 3 [3–16] years. There was a female predominance with a sex ratio of 0.42. The majority of patients had secondary education (52%). The distribution of the different subtypes was dominated by the oligoarticular form (30%) and the enthesitis-related arthritis form (26%), followed by the polyarticular FR + (*n* = 1), polyarticular FR- (*n* = 3), psoriatic arthritis (*n* = 3), systemic (*n* = 1) and undifferentiated (*n* = 1) forms. The mean physical activity score assessed by the Oxford score was 73.52 ± 35.8 [0 – 100]. Plantar footprint abnormalities of the hollow and flat foot were found in 39% and 30% of cases respectively. These abnormalities were unilateral and bilateral in 7 and 9 patients respectively. Eleven patients (48%) had a hindfoot abnormality and 30% of them had a limitation of joint movement range. There was no statistically significant association between the physical domain of the Oxford score and the presence of a limited range of the talocrural joint (*p*> 0.05). A significant reduction in physical activity was associated with hindfoot pain (5.37 *vs* 89.56; *p*< 0.001) but was not associated with the presence of hindfoot abnormalities (*p* = 0.05). The presence of flat feet was significantly associated with impairment in all domains of the Oxford score, particularly in the physical domain (36.79 for the flat foot group *vs* 89.2 for the group without flat feet, *p* = 0.001). However, the presence of a hollow foot did not lead to an alteration of the physical activity of JIA patients (*p*> 0.05).

**Conclusion:**

Our study showed that the flat foot was associated with a reduction in physical activity. Early detection of this abnormality and adapted podiatric care could improve the function of young patients with JIA.

**References**

[1] Gueddari S, Amine B, Rostom S, *et al.* Physical activity, functional ability, and disease activity in children and adolescents with juvenile idiopathic arthritis. Clin Rheumatol. 2014; 33(9):1289–1294.

The implication to policy, practice, research and advocacy

It is important to ensure that children with juvenile idiopathic arthritis are receiving appr

